# Pre-procedural vitamin D deficiency and poor prognosis post-thrombectomy in patients with acute anterior circulation large vessel occlusion: a retrospective cohort study

**DOI:** 10.3389/fneur.2026.1717442

**Published:** 2026-02-11

**Authors:** Pan Xu, Kewei Liu, Jinyu Li, Wuying Li, Weihua Deng, Huameng Huang, Xing Li

**Affiliations:** 1 Department of Neurology, School of Medicine, The Second Affiliated Hospital of South China University of Technology (Guangzhou First People's Hospital), Guangzhou, China; 2 Department of Interventional Medicine, School of Medicine, The Second Affiliated Hospital of South China University of Technology (Guangzhou First People's Hospital), Guangzhou, China

**Keywords:** C-reactive protein, delayed recanalization, ischemic stroke, mechanical thrombectomy, vitamin D deficiency

## Abstract

**Background:**

Vitamin D deficiency is associated with poor prognosis of stroke. However, its role after mechanical thrombectomy (MT) in acute ischemic stroke (AIS) remains unclear. This study aimed to explore the relationship between pre-procedural vitamin D levels and the clinical prognosis of AIS patients after MT.

**Methods:**

A total of 113 AIS patients with successful MT were enrolled in this study and their clinical data were analyzed. Patients were divided into two groups based on their 3-month modified Rankin Scale scores (mRS: 0–2 vs. 3–6). Predictors of adverse prognosis were determined using multivariate logistic regression analysis. In order to evaluate nonlinear associations and mortality risk, restricted cubic spline (RCS) models, receiver operating characteristic (ROC) curve analysis, and Kaplan–Meier survival curve analysis were used.

**Results:**

In the patient cohort, compared to the favorable outcome group (mRS 0–2; 31%), patients with poor outcomes (mRS 3–6; 69%) exhibited significantly lower pre-procedural vitamin D levels (14.72 vs. 28.38 ng/mL). Multivariate logistic regression analysis identified severe vitamin D deficiency, delayed recanalization, and elevated high-sensitivity C-reactive protein as independent risk factors. RCS analysis revealed a nonlinear threshold effect of vitamin D on prognosis, with an inflection point at 20 ng/mL. Patients with severe vitamin D deficiency (<10 ng/mL) exhibited markedly higher 180-day mortality compared to those with normal vitamin D levels (≥30 ng/mL) (38.1% vs. 5.0%).

**Conclusion:**

Pre-procedural vitamin D deficiency is independently associated with poor functional outcomes and increased mortality after successful MT. Our findings highlight the potential prognostic value of vitamin D assessment in this patient population. Future interventional studies are needed to determine whether correcting vitamin D deficiency could optimize post-thrombectomy management.

## 1 Introduction

Acute ischemic stroke (AIS) remains a leading cause of death and disability worldwide (1), and emergency mechanical thrombectomy (MT) has become a core approach in treating large vessel occlusion (2). Clinical studies indicate that despite the continuous advancements in endovascular treatment techniques, only approximately 45% of patients who successfully achieved vascular recanalization [modified thrombolysis in cerebral infarction (mTICI) ≥ 2b)] reached functional independence [modified Rankin Scale (mRS) < 2] within 90 days after the procedure (3). Moreover, approximately 50% of patients exhibited a poor prognosis or even died (4, 5). This discrepancy underscores the urgent need to identify modifiable prognostic factors beyond procedural success.

Vitamin D has attracted increasing attention as a potential neuroprotective agent (6). Preclinical studies have shown that vitamin D plays an important role in regulating neuroinflammation, maintaining the integrity of the blood–brain barrier, and promoting neuronal survival after ischemia (7–10). A prospective study investigating the relationship between vitamin D and the 5-year outcomes of stroke patients revealed that the serum levels of 25-hydroxyvitamin D [25(OH)D] in patients with a good prognosis were significantly higher than those in patients with a poor prognosis (11). Epidemiological evidence further indicates that the lower serum 25(OH)D levels are independently associated with medium- and long-term stroke recurrence and mortality in patients with ischemic stroke (10, 12). Nevertheless, the specific prognostic potential of pre-procedural vitamin D status in the context of MT remains limited.

The serum 25(OH)D levels ≥75 nmol/L (≥30 ng/mL) are generally considered adequate for overall health: on the other hand, its levels between 50 and 75 nmol/L (20–30 ng/mL) indicate “insufficiency,” and the levels <50 nmol/L (<20 ng/mL) indicate “deficiency” (13). Vitamin D deficiency and insufficiency are a global health concern, affecting more than one billion children and adults worldwide (14), and are also common in patients with stroke (15). The pleiotropic effects of vitamin D on vascular function, oxidative stress, thrombosis, and neural repair mechanisms suggest that it may affect the recovery process after recanalization therapy (9, 16, 17). Importantly, vitamin D status is rapidly quantifiable and correctable, making it as an ideal biomarker for personalized post-thrombectomy management.

We hypothesized that lower pre-procedural serum vitamin D levels are independently associated with poorer functional outcomes after successful mechanical thrombectomy. Therefore, the primary objective of this study was to investigate the association between pre-procedural serum 25(OH)D levels and 3-month functional outcomes in AIS patients with acute anterior circulation large vessel occlusion undergoing MT with successful recanalization.

## 2 Data and methods

### 2.1 Study design and participants

This is a single-center, retrospective cohort study. A total of 113 eligible patients were included in the final analysis. The study was approved by the medical ethics committee of Guangzhou First People’s Hospital (approval number: K-2024-139-01).

### 2.2 Data collection and vitamin D measurement

#### 2.2.1 Data collection

Demographic, clinical, laboratory, procedural, and outcome data were collected from the electronic medical records. The collected data included age, sex, vascular risk factors, pre-stroke mRS, admission National Institutes of Health Stroke Scale (NIHSS) and Glasgow Coma Scale (GCS) scores, stroke etiology (TOAST classification), time metrics (onset-to-puncture, puncture-to-recanalization), number of thrombectomy attempts, successful recanalization status (mTICI≥2b), and routine laboratory parameters [including high-sensitivity C-reactive protein (hs-CRP), B-type natriuretic peptide (BNP), D-dimer, serum calcium, lipid profile, and complete blood count]. The primary outcome was functional status assessed by the modified Rankin Scale (mRS) at 3 months post-procedure.

#### 2.2.2 Vitamin D measurement

Serum 25-hydroxyvitamin D [25(OH)D] levels were measured pre-procedurally at the emergency department admission or immediately before the angiography procedure, prior to anesthesia or arterial puncture. The measurement was performed using a chemiluminescence immunoassay (CLIA) on the Siemens ADVIA Centaur XP platform. The results are reported in ng/mL. According to the assay reference range and clinical guidelines, levels were categorized as: deficiency (<20 ng/mL), insufficiency (20–30 ng/mL), and sufficiency (≥30 ng/mL).

### 2.3 Research methods

The basic clinical data of AIS patients with anterior circulation infarction and successful recanalization of MT during hospital stay and follow-up were collected. The primary outcome event was poor prognosis (mRS ≥ 3). After 90 and 180 days of discharging the patients, neurologists who were blinded to the patients’ vitamin D levels and surgical details conducted standardized telephonic follow-ups to evaluate the neurological function recovery of the patients and the occurrence of death during this period according to the mRS Scale. The discrepancies were resolved by consensus.

### 2.4 Outcome assessment

The primary targeting outcome was assessed using the mRS on follow-up. A previous study defined poor outcome as an mRS value of >2 (18). Therefore, in the current study, mRS was defined as a dichotomous variable: an mRS score of 0–2 range indicated no symptoms to a minimal disability and reflected a good outcome, while an mRS score of 3–6 range indicated moderate to severe disability or death, reflecting a poor outcome. This study specifically aimed to assess the association between pre-procedural vitamin D levels and these post-thrombectomy functional outcomes.

### 2.5 Statistical analyses

In this study, SPSS 27.0 and R software version 4.2.3 were used for statistical analyses. The statistically significant indicators were obtained using the univariate binary logistic regression analysis and included in the multivariate binary logistic regression analysis to obtain independent predictors with significant influence. Based on the adjusted odds ratio (OR), the relationship between pre-procedural vitamin D levels and adverse outcomes was analyzed using OR and 95% confidence interval (CI). The RCS regression model was used to explore the relationship between pre-procedural vitamin D levels and prognosis; inflection points were identified via likelihood ratio tests. The receiver operating characteristic (ROC) curve was further applied to analyze the diagnostic value of vitamin D for adverse outcomes. In addition, all the statistical tests were two-sided, and a *p*-value of <0.05 defined statistical significance. Missing data were not included in the relevant analysis, and no data replacement processing was performed. Missing data were minimal and sporadic across variables (e.g., specific laboratory values in <5% of cases). These cases were excluded from the respective analyses involving the missing variables. Given the small proportion and the lack of evidence suggesting these missing data were non-random, their exclusion is unlikely to have introduced substantial bias or altered the primary conclusions of the study. In all regression analyses, the binary outcome variable was coded as: poor functional outcome (mRS 3–6) = 1, favorable outcome (mRS 0–2) = 0. For continuous predictors, odds ratios (OR) and hazard ratios (HR) < 1 indicate a protective effect (decreased risk with increasing values), while values >1 indicate a risk factor (increased risk with increasing values).

## 3 Results

### 3.1 Patient characteristics

**Figure 1:**
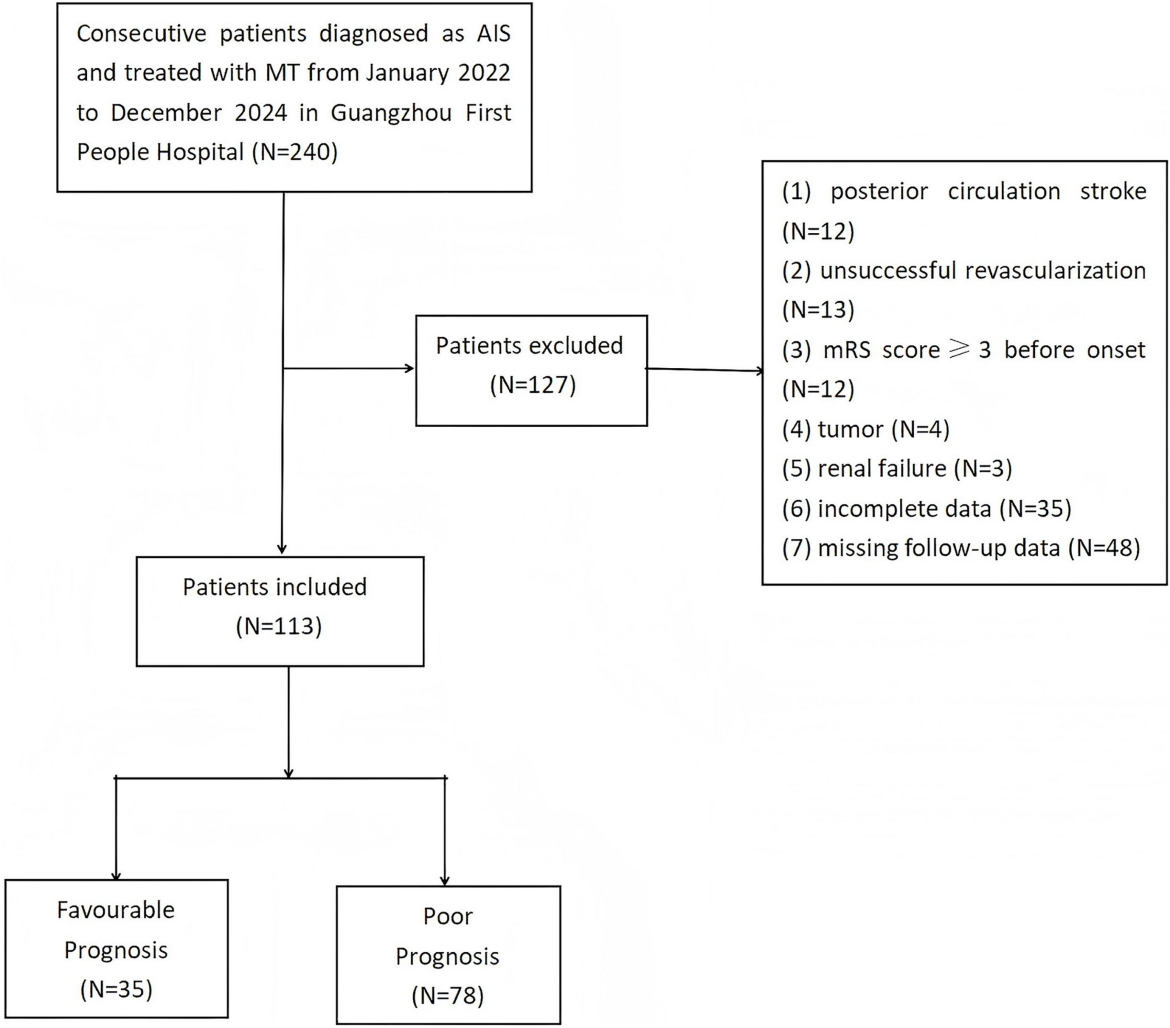
Flowchart of inclusion and exclusion.

Figure 1 illustrates the participant selection flowchart. The inclusion criteria were as follows: (1) age ≥18 years; (2) AIS confirmed using neuroimaging (anterior circulation large vessel occlusion involving the internal carotid artery or middle cerebral artery M1/M2 segments); (3) MT performed within 24 h of symptom onset; (4) successful post-MT recanalization (mTICI ≥2b); and (5) preprocedural 25(OH)D measurement was available. The exclusion criteria were as follows: (1) posterior circulation stroke; (2) other serious neurological diseases, such as dementia and advanced Parkinson’s disease; (3) life expectancy <6 months; (4) mRS ≥ 3 points before stroke; (5) vitamin D metabolic diseases (abnormal parathyroid function and stage 3 or above chronic kidney disease); (6) recent vitamin D supplementation (≤3 months); (7) incomplete clinical data; and (8) lost to follow-up or withdrawal of consent.

Among the 113 AIS patients included in this study who underwent MT and achieved successful vascular recanalization, 78 (69.0%) exhibited a poor prognosis (defined as mRS 3–6) at 3 months (Table 1). As compared to those with better prognosis, the proportion of male patients was significantly lower in the poor prognosis group (*p* = 0.011) and significantly higher pre-procedural NIHSS and GCS scores (*p* < 0.001). During procedure, the patients with poor prognosis underwent significantly more thrombectomy sessions (*p* < 0.001) with longer times from onset to puncture (*p* < 0.001) compared to those with a good prognosis. Moreover, the patients with poor prognosis had significantly lower pre-procedural levels of vitamin D, Ca^2+^, triglycerides, hemoglobin, and red blood cell count compared to those with good prognosis (*p* < 0.05). Furthermore, the levels of hs-CRP, BNP, D-dimer, and the ratio of neutrophils to lymphocytes were higher in the poor prognosis group (*p* < 0.05). There were no significant differences in other clinical data and laboratory parameters between the groups. Based on the pre-procedural vitamin D levels, 62 patients (54.9%) were categorized as deficient (<20 ng/mL), 31 (27.4%) as insufficient (20–30 ng/mL), and 20 (17.7%) as sufficient (≥30 ng/mL).

**Table 1:** Baseline characteristics of the two groups with different prognosis (based on 3-month mRS).

Variable	mRS 3–6 (n = 78)	mRS 0–2 (n = 35)	*p*-value
Age	73 (60–82)	67 (60–75)	0.105
Male	39 (50.0)	27 (77.1)	0.011
Pre-procedural ^1^NIHSS Score	15 (10–20)	9 (7–14)	<0.001
Pre-procedural ^2^GCS Score	9 (6–12)	13 (10–15)	<0.001
Medical history			
Hypertension	43 (55.1)	23 (65.7)	0.191
Diabetes	13 (16.7)	9 (25.7)	0.475
Coronary heart disease	16 (20.5)	6 (17.1)	0.748
Atrial fibrillation	21 (26.9)	5 (14.3)	0.169
Stroke	16 (20.5)	3 (8.6)	0.136
Smoke	13 (16.7)	7 (20.0)	0.598
Drink	9 (11.5)	6 (17.1)	0.379
Occluded blood vessel			
Internal carotid	35 (44.9)	12 (34.3)	0.191
Anterior cerebral artery	4 (5.1)	3 (8.6)	0.460
Middle cerebral artery	51 (65.4)	20 (57.1)	0.654
Multivessel	13 (16.7)	2 (5.7)	0.143
^3^TOAST classification			0.019
LAA	49 (62.8)	30 (85.7)	
CE	29 (37.2)	5 (14.3)	
Procedure process			
Local anesthesia	59 (75.6)	32 (91.4)	0.061
Intravenous thrombolysis	21 (26.9)	12 (34.3)	0.629
Number of thrombectomy	2 (1–3)	1 (1–2)	<0.001
Time from onset to recanalization (min)	333 (260–435)	203 (178–290)	<0.001
Laboratory test index			
Vitamin D (ng/mL)	14.72 (9.52–21.28)	28.38 (22.31–35.62)	<0.001
Ca^2+^ (mmol/L)	2.12 ± 0.16	2.20 ± 0.13	0.012
Alkaline phosphatase (U/L)	72 (57–85)	66 (55.5–79.0)	0.318
Glycated hemoglobin (%)	6.0 (5.6–6.5)	5.9 (5.6–6.3)	0.768
HCY (μmol/L)	10.83 (8.03–13.31)	12.01 (8.38–13.61)	0.388
LDL (mmol/L)	2.59 ± 0.99	2.66 ± 0.98	0.713
HDL (mmol/L)	0.99 (0.87–1.22)	0.92 (0.84–1.18)	0.401
Total cholesterol (mmol/L)	3.95 ± 1.34	4.07 ± 1.37	0.696
Triacylglycerol (mmol/L)	0.99 (0.73–1.42)	1.10 (1.00–1.96)	0.021
Uric acid (μmol/L)	362 ± 156	345 ± 124	0.576
Creatinine (μmol/L)	81 (66–109)	78 (69–88)	0.452
hs-CRP (mg/L)	8.4 (3.9–17.7)	5.6 (2.7–9.9)	0.027
Cystatin C (mg/L)	0.97 (0.72–1.32)	0.88 (0.78–1.28)	0.757
BNP (pg/mL)	1,130 (377–2,723)	487 (157–891)	0.015
D-Dimer (μg/L)	2,200 (910–3,410)	1,020 (350–1765)	0.003
Neutrophil to lymphocyte ratio	8.17 (4.01–13.97)	5.71 (3.13–7.44)	0.031
Hemoglobin (g/L)	126 ± 19	136 ± 17	0.014
Fibrinogen (g/L)	3.17 (2.66–3.73)	3.17 (2.80–3.46)	0.771
Erythrocyte (10^12^/L)	4.26 (3.69–4.68)	4.58 (4.12–4.92)	0.035
Thrombocyte (10^9^/L)	199 ± 66	215 ± 60	0.290

Min, minute.

1 NIHSS Score, National Institute of Health Stroke Scale.

2 GCS Score, Glasgow Coma Scale.

3 TOAST classification, Trial of org 10172 in acute stroke treatment.

### 3.2 Correlation between pre-procedural serum vitamin D level and adverse outcomes after thrombectomy and recanalization

After identifying the significant correlation factors through initial screening using the univariate binary logistic regression analysis, the variables and gender were further included in the multivariate binary logistic regression model. The multivariable Model II was adjusted for sex, NIHSS score, GCS score, thrombectomy attempts, recanalization time, TOAST classification, and laboratory markers (Ca^2+^, hs-CRP, BNP, etc.). It identified two independent risk factors, including vitamin D deficiency and delayed recanalization. Each 1 ng/mL increase in vitamin D was associated with a 12.1% reduction in poor outcome risk (aOR, 0.879; 95% CI, 0.809–0.956; *p* = 0.003). Delayed recanalization: Per minute delay, the risk of poor prognosis increased by 1.1% (aOR, 1.011; 95% CI 1.003–1.019; *p* = 0.005). The results of multivariate binary logistic regression are shown in Table 2.

**Table 2:** Univariate and multivariate binary logistic regression analysis of pre-procedural vitamin D and poor prognosis (based on 3-month mRS).

Variable	Model I:		Model II:	
	OR (95%CI)	Model I: *p*-value	aOR (95%CI)	Model II: *p*-value
Male	0.316 (0.127–0.782)	0.013		
Pre-procedural NIHSS Score	1.164 (1.070–1.267)	<0.001		
Pre-procedural GCS Score	0.826 (0.734–0.928)	<0.001		
TOAST classification	3.364 (1.173–9.648)	0.024		
Number of thrombectomy	3.265 (1.659–6.425)	<0.001		
Time from onset to recanalization (min)	1.012 (1.006–1.017)	<0.001	1.011 (1.003–1.019)	0.005
Vitamin D	0.879 (0.833–0.928)	<0.001	0.879 (0.809–0.956)	0.003
Ca^2+^	0.026 (0.001–0.489)	0.015		
Triacylglycerol	0.716 (0.501–1.023)	0.693		
hs-CRP	1.055 (1.001–1.111)	0.045		
BNP	1.000 (1.000–1.001)	0.055		
D-Dimer	1.000 (1.000–1.000)	0.107		
Neutrophil to lymphocyte ratio	1.098 (1.014–1.189)	0.021		
Hemoglobin	0.971 (0.948–0.995)	0.018		
Erythrocyte	0.504 (0.274–0.927)	0.028		

Model I: Confounding factors were not adjusted.

Model II: Multivariate logistic regression adjusted for sex, NIHSS, GCS, number of thrombectomy, recanalization time, TOAST classification, Ca^2+^, hs-CRP, BNP, D-Dimer, neutrophil to lymphocyte ratio, and hemoglobin. OR>1 indicates increased odds of poor outcome (mRS 3–6); OR<1 indicates decreased odds of poor outcome.

### 3.3 Pre-procedural serum vitamin D levels and adverse outcomes showed a nonlinear relationship after thrombectomy and recanalization

**Figure 2:**
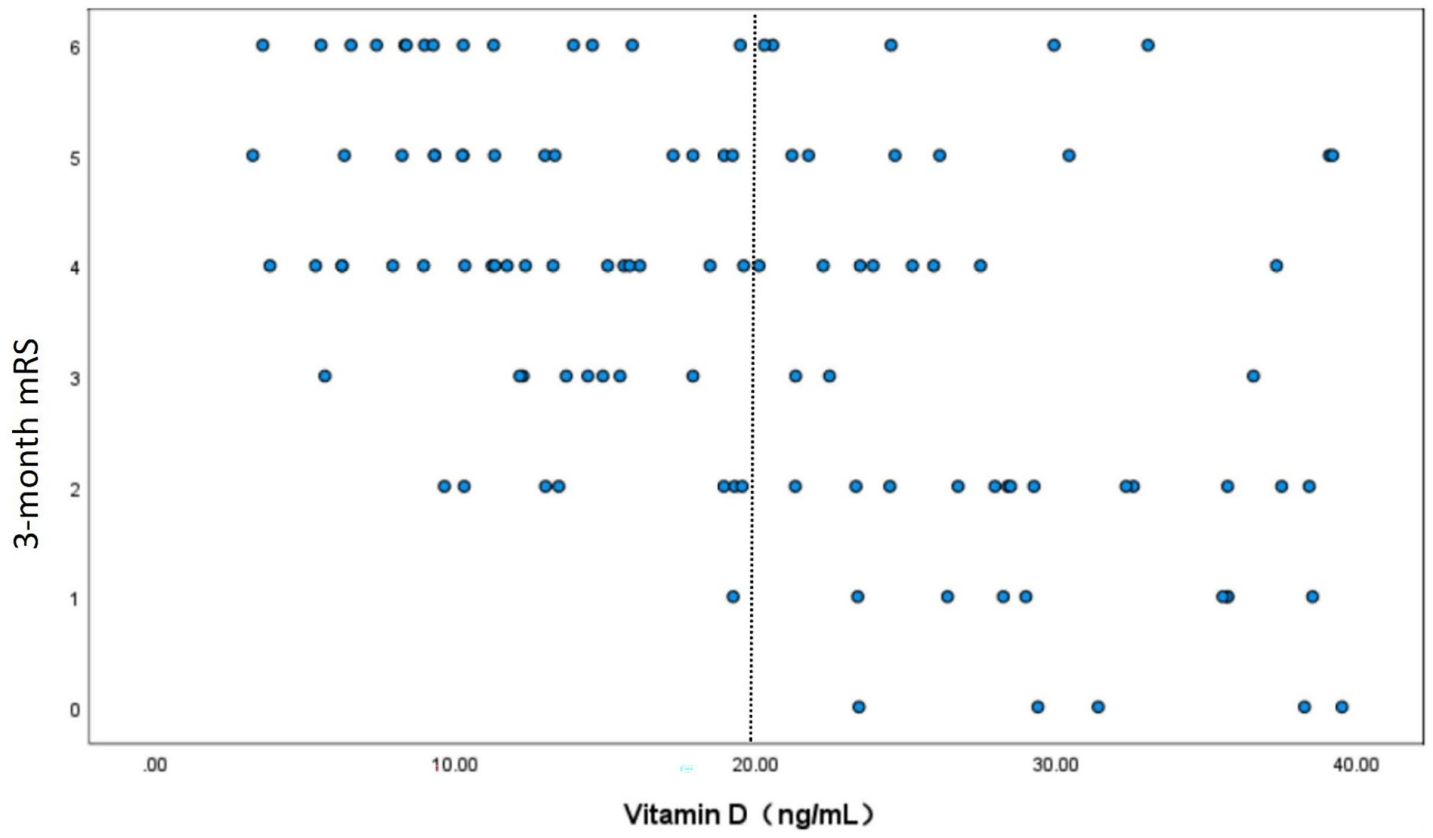
This figure shows the scatter plots of serum vitamin D levels and 3-month modified Rankin Scale (mRS) scores in the study cohort. Each point represents the data of a patient, where the horizontal axis is the serum vitamin D level (ng/mL) and the vertical axis is the 3-month mRS core. The Spearman correlation coefficient *r* = −0.472 (*p* < 0.001), indicating a significant negative correlation between the two; that is, the higher the concentration of vitamin D, the lower the mRS core (the better the prognosis of neurological function). The distribution of data points shows a trend of decreasing mRS core with the increase of vitamin D level. However, when the vitamin D concentration is lower than 20 ng/mL, the variability of data points increases, suggesting the possible existence of a threshold effect.

A scatter plot demonstrated a significant negative relationship between serum vitamin D levels and 3-month mRS scores in the study cohort, with higher vitamin D levels associated with lower (better) mRS outcomes (Spearman’s *r* = −0.472, *p* < 0.001). The distribution of data points suggested a monotonic trend; however, variability increased at lower vitamin D levels (<20 ng/mL), potentially indicating threshold effects (Figure 2).

**Figure 3:**
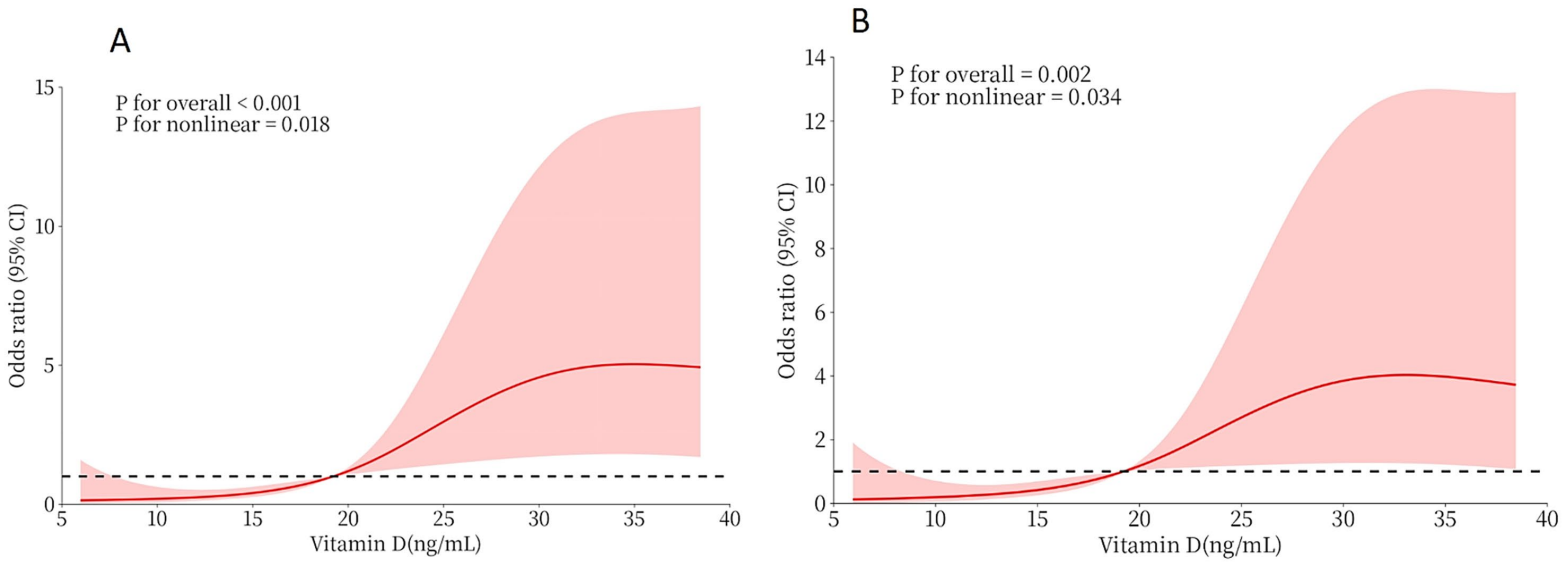
**(A)** The RCS curve of the association between serum vitamin D levels and prognosis among all participants. **(B)** The gender, the number of thrombectomy, the pre-procedural NIHSS score, and the pre-procedural GCS score were adjusted.

**Figure 4:**
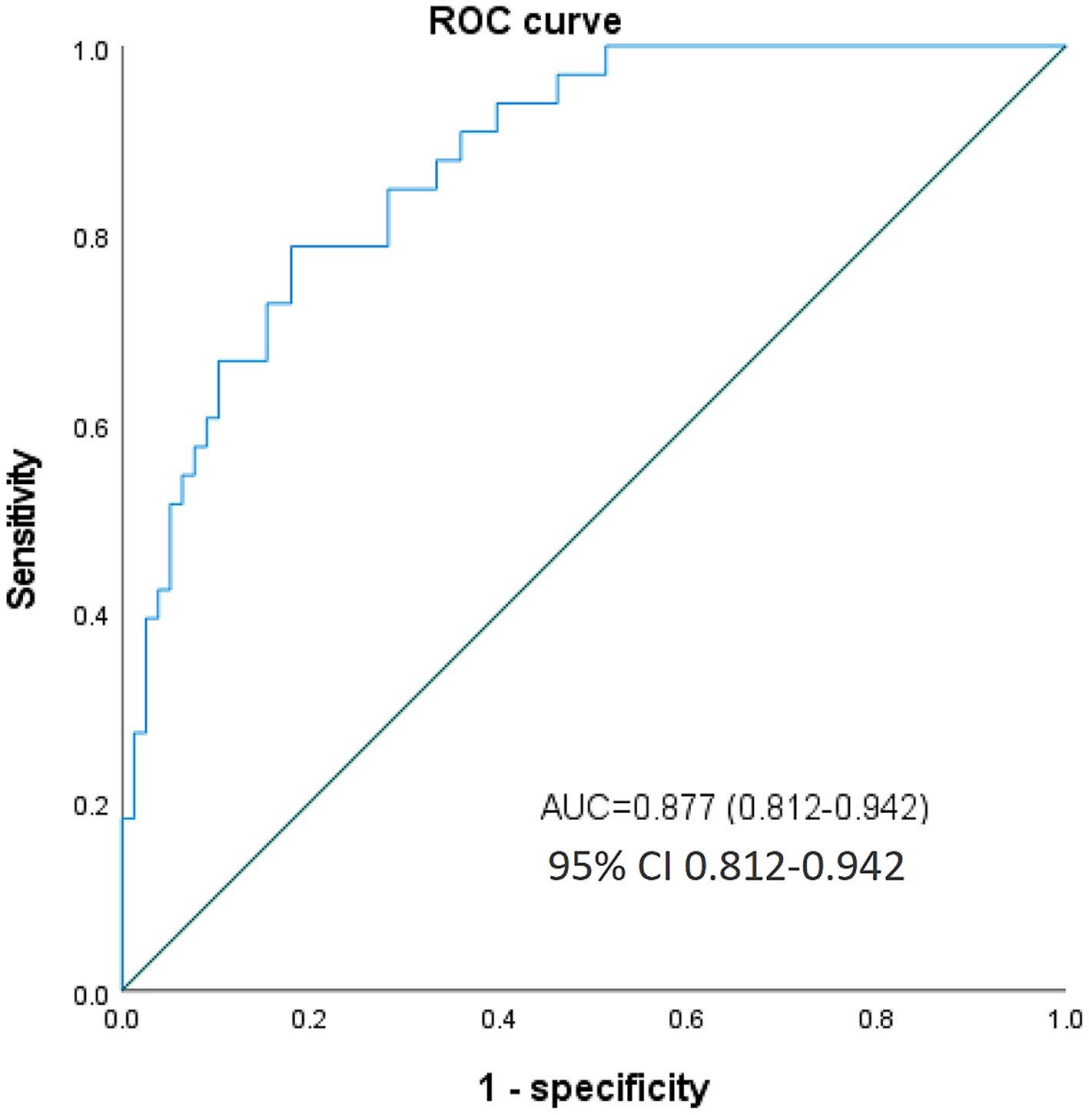
ROC curve for evaluating the prognostic predictive ability of vitamin D, AUC = 0.877 (95% CI 0.812–0.942), sensitivity was 73.1% and specificity was 82.9%.

RCS analyses were performed to further investigate the relationship between pre-procedural vitamin D levels and medium-to-long-term functional outcomes in AIS patients following successful MT. The unadjusted RCS model revealed a significant nonlinear correlation between vitamin D levels and poor prognosis (mRS 3–6) (*p*-value for nonlinear = 0.018). This nonlinearity persisted after adjusting for sex, number of thrombectomy attempts, pre-procedural NIHSS score, and GCS score (*p*-value for nonlinear = 0.034) (Figure 3). The inflection point was identified at 20 ng/mL using likelihood ratio tests. Below this threshold, lower vitamin D levels were strongly associated with worse outcomes, while the association plateaued above 20 ng/mL. ROC analysis confirmed the prognostic potential of vitamin D, with an optimal cut-off value of 20 ng/mL, yielding an AUC value of 0.877 (0.812–0.942). At this threshold, sensitivity was 73.1% and specificity was 82.9% (Figure 4).

### 3.4 Correlation between serum vitamin D levels and the risk of death after MT in AIS patients

**Figure 5:**
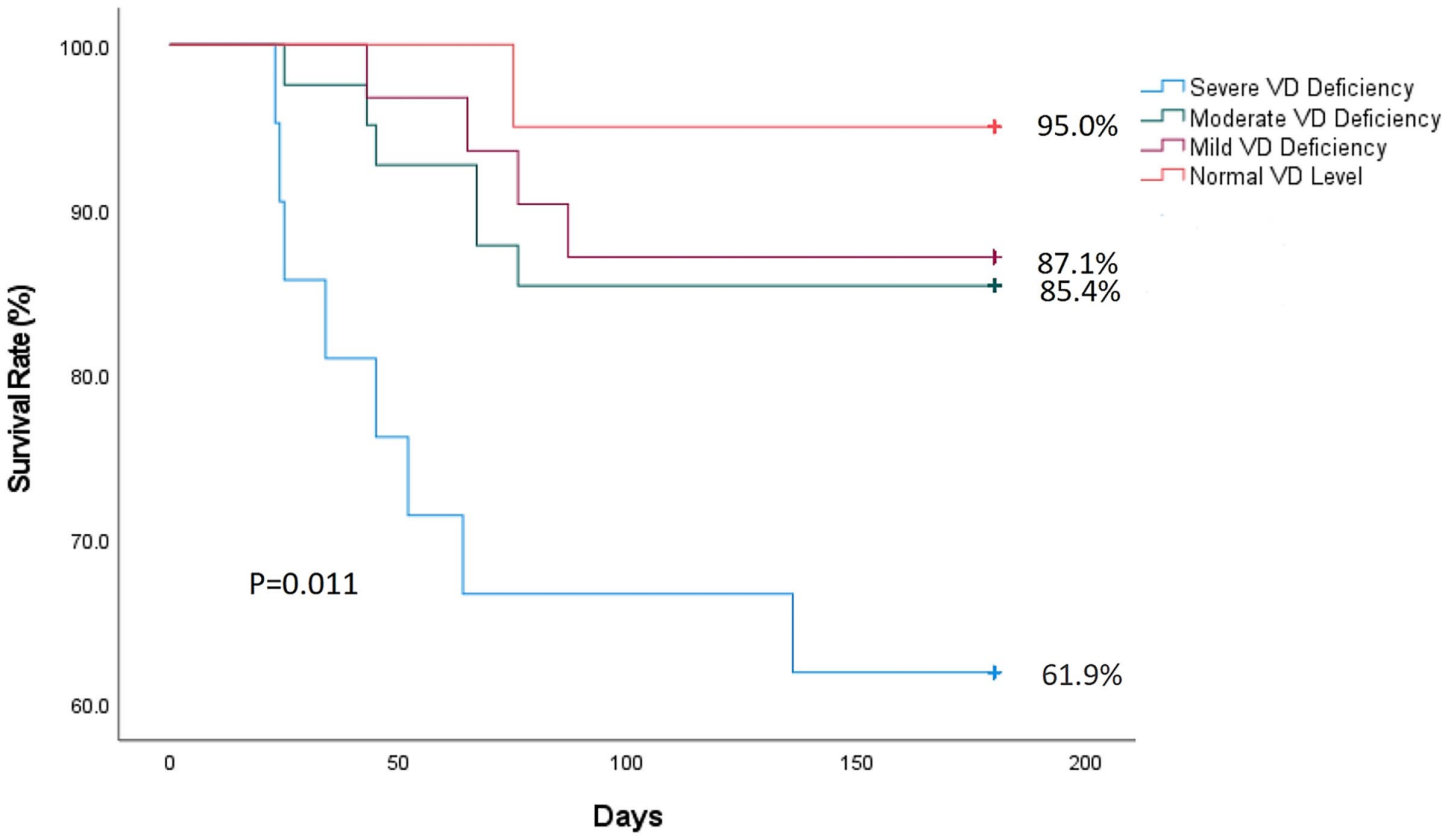
Kaplan–Meier survival curve analysis, stratified by vitamin D level, severe vitamin D deficiency (<10 ng/mL), moderate vitamin D deficiency (10–20 ng/mL), mild vitamin D deficiency (20–30 ng/mL), and normal vitamin D levels (≥30 ng/mL).

To further explore the association between vitamin D levels and long-term prognosis, we analyzed the relationship between vitamin D levels and 180-day mortality. After a 6-month follow-up, 19 patients (16.8%) died, which were divided into four groups based on the vitamin D level. A total of 8 (38.1%), 6 (14.6%), 4 (12.9%), and 1 (5%) patients belonged to group 1 [severe vitamin D deficiency (<10 ng/mL)], group 2 [moderate vitamin D deficiency (10–20 ng/mL)], group 3 [mild vitamin D deficiency (20–30 ng/mL)], and group 4 [normal vitamin D (≥30 ng/mL)], respectively. Figure 5 shows that the individuals with lower vitamin D levels exhibited a higher risk of death at 90 days and 180 days. Severe deficiency (<10 ng/mL) had significantly higher 180-day mortality (38.1% vs. 5%, *p* = 0.011). This is consistent with the Cox proportional hazards model showing that each 1 ng/mL increase in vitamin D was associated with a 6.4% reduction in mortality risk (HR = 0.936; 95% CI: 0.886–0.989).

## 4 Discussion

This study systematically explored for the first time the relationship between pre-procedural vitamin D status and functional prognosis after 3 months of MT in patients with acute anterior circulation large vessel occlusion. The main findings of this study can be summarized in three aspects. (1) Vitamin D deficiency (<20 ng/mL) is independently associated with poor 3-month functional prognosis. After adjusting for stroke severity and procedural parameters, the risk of poor prognosis decreased by 12.1% for every 1 ng/mL increase in 25(OH)D level (aOR = 0.879). (2) Obvious threshold effects were found. When 25(OH)D was lower than 20 ng/mL, the deterioration risk of the 3-month mRS score increased sharply. (3) The 180-day mortality rate of the severe vitamin D deficiency group (<10 ng/mL) was 7.6 times that of the normal group (38.1% vs. 5.0%). These results provide new biological markers for predicting clinical prognosis and pointing out potential directions for targeted intervention research.

The results of this study were consistent with those of the previous observational studies; however, this study provided more accurate threshold data. Some clinical studies (17, 19–22) reported an association between vitamin D deficiency and poor prognosis of stroke; however, they did not evaluate the status of vascular recanalization. By strictly limiting the patient population with mTICI≥2b, the current study confirmed for the first time that even in the case of successful recanalization, vitamin D status was significantly associated with the recovery of neurological function. This finding might partially explain the common clinical phenomenon of “ineffective recanalization.” In this study, approximately 76.3% of the patients with low vitamin D failed to achieve functional independence despite vascular recanalization. Previous clinical studies (23–25) have also confirmed the positive impact of vitamin D supplementation on specific outcomes in stroke patients, such as improved functional recovery scores and reduced mortality. There are three possible mechanisms by which vitamin D levels might affect the prognosis of stroke. (1) Neuroprotective effect: Vitamin D exerts neuroprotective effects by weakening hiprotein-induced pyroptosis and ferroptosis, thereby alleviating neuroinflammation, reducing oxidative stress injury, and protecting the survival of neurons in the ischemic penumbra (9, 26). (2) Vascular function regulation: vitamin D deficiency might interact with hyperandrogenemia, exacerbating microcirculation disorders after recanalization (27). Meanwhile, vitamin D can cause vascular endothelial dysfunction and thickening of the vascular wall, thereby affecting the normal function of cerebral blood vessels and increasing the risk of stroke and cognitive impairment (9). (3) Systemic inflammation regulation: vitamin D regulate the production of endothelial CXCL10, inhibit the production capacity of IFN-*γ*, reduce the level of IL-6, decrease the release of inflammatory factors, and control the occurrence of inflammation (8, 9). The current study found that elevated levels of hs-CRP showed a borderline significant positive association with poor prognosis in the multivariable model (aOR = 1.055, 95% CI: 1.001–1.111). This suggests the involvement of inflammatory pathways in stroke outcomes. In addition, the association between vitamin D and calcium metabolism [low calcium levels are associated with poor prognosis (28)] might also affect neuroelectrophysiological stability (7).

The current study used RCS analysis and determined the key threshold of 20 ng/mL for 3-month functional outcome. This value is lower than the current bone health standard recommended by the Endocrinology Society (30 ng/mL) (29). However, this value is close to that proposed by recent studies. A 25 (OH) D level >55 nmol/L (22 ng/mL) was associated with a reduced mortality rate among stroke survivors (25). It was worth noting that when the 25(OH)D level was 20 ng/mL, further increase did not bring additional benefits for functional outcome, suggesting the possible existence of a “ceiling effect.” This might have a significant guiding value for clinical practice. On the one hand, 20 ng/mL is more feasible as the intervention critical point. On the other hand, excessive supplementation might not be necessary. However, it should be noted that this study was an observational design. In the future, randomized controlled trials (RCTs) will be needed to verify whether vitamin D supplementation can improve prognosis.

This study has several limitations. First, as a single-center observational study, there may be selection bias, despite adopting consecutive case inclusion criteria. Second, although vitamin D testing was carried out in standardized laboratories, the sampling time was not unified, and the difference in sampling times (day and night) might affect the results. Third, potential confounding factors, such as vitamin D binding protein (VDBP) gene polymorphism and sunlight exposure duration, were not included. Fourth, the causal association of intervention data lacking vitamin D supplementation therapy needs to be confirmed through RCTs.

Our findings suggest several avenues for future research. (1) The 25(OH)D level should be routinely tested for all AIS patients scheduled for MT. (2) For patients with vitamin D < 20 ng/mL, exploring supplementary therapy in clinical trials may be warranted (although the optimal dose and administration timing require further study). (3) Vitamin D status should be incorporated into prognostic prediction models (such as in combination with HIAT scores). Future studies should focus on addressing three issues: (1) the safety and efficacy of intravenous vitamin D supplementation in the hyperacute phase (<6 h), (2) the interaction between vitamin D and different thrombectomy techniques, and (3) the protective effect of long-term maintenance treatment on neurological function.

The current study findings proposed a clinically actionable threshold (20 ng/mL) for vitamin D supplementation in MT patients. A randomized trial is urgently needed to test whether rapid correction of deficiency (<24 h post-MT) could improve outcomes.

## 5 Conclusion

This study revealed for the first time that pre-procedural vitamin D deficiency was independently associated with poor functional prognosis and increased risk of death after MT in AIS patients, with a clear dose-effect relationship. These findings highlight the potential value of vitamin D as a prognostic biomarker and suggest the need to investigate early screening and correction of vitamin D deficiency in MT patients. Future studies should focus on exploring the underlying mechanism and conducting interventional trials to further optimize potential neuroprotection strategy centered on vitamin D.

## Data Availability

The original contributions presented in the study are included in the article/Supplementary material, further inquiries can be directed to the corresponding authors.
